# Fluctuation Induces Evolutionary Branching in a Mathematical Model of Ecosystems

**DOI:** 10.1371/journal.pone.0003925

**Published:** 2008-12-12

**Authors:** Masashi Tachikawa

**Affiliations:** Complex Systems Biology Project, Exploratory Research for Advanced Technology (ERATO), Japan Science and Technology Agency (JST), Tokyo, Japan; Center for Genomic Regulation, Spain

## Abstract

Ecological systems are always subjected to various environmental fluctuations. They evolve under these fluctuations and the resulting systems are robust against them. The diversity in ecological systems is also acquired through the evolution. How do the fluctuations affect the evolutionary processes? Do the fluctuations have direct impact on the species diversity in ecological systems? In the present paper, we investigate the relation between the environmental fluctuation and the evolution of species diversity with a mathematical model of evolutionary ecology. In the model, individual organisms compete for a single restricted resource and the temporal fluctuation in the resource supply is introduced as the environmental fluctuation. The evolutionary process is represented by the mutational change of genotypes which determines their resource utilization strategies. We found that when the environmental state is switched form static to fluctuating conditions, the initial closely related population distributed around the genotype adapted for the static environment is destabilized and divided into two groups in the genotype space; i.e., the evolutionary branching is induced by the environmental fluctuation. The consequent multiple species structures is evolutionary stable at the presence of the fluctuation. We perform the evolutionary invasion analysis for the phenomena and illustrate the mechanisms of the branchings. The results indicate a novel process of increasing the species diversity via evolutionary branching, and the analysis reveals the mechanisims of the branching preocess as the response to the environmental fluctuation. The robustness of the evolutionary process is also discussed.

## Introduction

Fluctuation is ubiquitous in nature. Biological systems are always exposed to temporal fluctuations of the environment. Hence, the systems have evolved under these fluctuations, and the acquired biological functions work at the presence of them. Recently, the relations between fluctuations and functions of biological systems are frequently studied [1], [2], [3], [4]. Ecological systems are no exception. Diversity, which is one of the most essential properties in ecology, has also evolved in the presence of fluctuations. The association between diversity and temporal fluctuation has been discussed, and concepts such as intermediate disturbance hypothesis [5], [6], storage effect [7] and homeochaos [8], which all represent the positive effects of the fluctuation on increase of the diversity, have been proposed.

In particular, the coexistence of multiple species under the fluctuated environment has been frequently investigated for rather simple ecological situations, in which the species and the environmental factors involved in the systems are identified [9], [10], [11]. The population dynamics of bacteria competing for a single resource in chemostat or ecological systems of phytoplankton are examples. Armstrong and McGehee [9] pointed out that in such situations, the temporal variation of populations invalidates the competitive exclusion principle, which insists that two species competing for the same resources cannot coexist [12]. Several studies on mathematical models [10], [11] demonstrated that the periodic fluctuations in environmental parameters promote the coexistence of two species competing for a single resource. The fluctuation in environment helps to maintain the species diversity.

Then, can the fluctuation induce the increase of species diversity? While the maintenance of species diversity concerns the stability in population dynamics, the increase of species diversity involves the evolutionary process. Although the autonomous periodic and chaotic behaviors in population dynamics were reported to induce the increase of species diversity [13], [14], [15], there is no direct demonstration of the increase of the species diversity as a response to the temporal fluctuation in environment. In this paper, we study a model of the evolutionary ecology in the presence of a temporal fluctuation, and demonstrate that the fluctuation induce the evolutionary branching of species. We also perform the evolutionary invasion analysis which reveals the mechanisms of the evolutionary branchings. These results and analysis highlight how fluctuation facilitates species diversity.

We consider the competition for a single restricted resource as the ecological situation. It is one of the simplest but most thoroughly investigated ecological situations [10], [16], and the competitive exclusion principle is applicable to it if the system is static. Therefore, the impact of environmental fluctuation on species diversity is characterized clearly by violation of the principle. In order to make a mathematical model, some characteristics of microbial ecosystems are referred in this study. This is because that the plenty of experimental evidences for microorganisms gives us the quantitative descriptions of their growth kinetics under the conditions of resource restriction. The Monod equation [17], [18], the empirical relation between growth rate and the amount of the restricted resource, is applicable for the supposed situation.

## Methods

**Figure 1:**
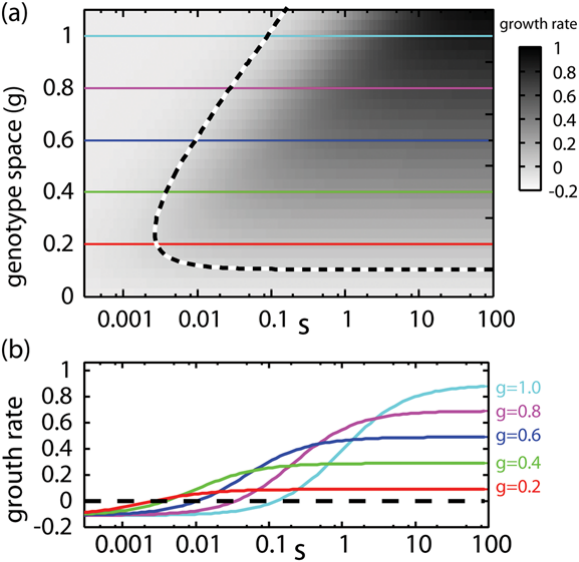
Monod equation. The growth rates determined by the Monod equation and the decay rate 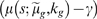 are displayed as a function of resource concentration *s* and genotype parameter *g*. The relation between genotype and paramters in the Monod equation is indicated in eq. (2). *γ* = 0.11 is adopted. (a) the growth rate is plotted with gray scales on *s*-*g* space. (b) Growth rate for genotypes *g* = 0.2,0.4,0.6,0.8,1.0 are plotted against *s*. The corresponding sections are indicated by lines with same colors in Fig. 1-a. In both figures, the zero growth rate is indicated by the dashed lines with black and white. The genotype with the fastest growth changes depending on *s*, which denotes the trade-off relation.

The Monod equation [17] is a saturation function to describe the growth rate of microorganisms limited by a resource,
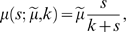
(1)where *s* denotes the concentration of the limited resource (Fig. 1). The two parameters, the maximum growth rate 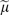 and the half-saturation constant *k*, are genetically determined and characterize the strategy for the resource utilization of the genotype. Note that this type of relation between resource concentration and growth rate is known as Holling's type II functional response to a resource concentration [19] in the general ecological theory. Therefore, our model and following results are not restricted to the microbial ecosystems but qualitatively same phenomena are expected in various ecological situations.

Kovárová-Kovar & Egli [18] shows how the parameters of the Monod equation change depending on the changes of the nutritional condition between copiotrophic and oligotrophic states. It implies that the parameters are not independently adjusted but are both determined by common traits. There are the positive correlation 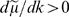, and the logarithmic relation 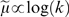 between parameters. The former denotes a trade-off relation (Fig. 1): fast growth in rich media (large 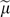) vs. the ability to grow in a wide range of resource concentrations (small *k*). Suppose that these relations reflect physical and chemical constraints, a genetic change of the resource utilization strategy is also restricted by the relations. Therefore, we adopt the above two relations as constraints for parameters, and introduce a continuous genetic parameter *g* which specifies the parameters of the Monod equation. We chose
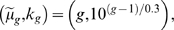
(2)with an appropriate scale conversion so that the range of dynamical changes of the genetic parameter are restricted in the region 0<*g*<1. The mutation indicates the small change in *g*. At the population level it is described with the diffusion process in *g* space.

Now we give the model equation. The population of individual genotype grows at its own growth rate 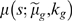 and decreases at the same rate *γ*, which stands the death and/or dilution from a supposed area. The resource *s* is supplied in a time-dependent manner which is represented by function *c*(*t*) and consumed by all populations in proportion to their growth rates. Let *x_g_* be the population density of a genotype, the model is given by a partial differential equation with globally interacting variable *s*,
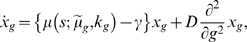
(3‐1)

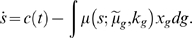
(3‐2)Partial differential term in equation (3-1) represents the population density change due to the mutation, and diffusion constant *D* is proportional to the mutation rate. The second term in the right hand side of the equation (3-2) represents the consumption of the resource. Since *x_g_* is the population density, the integration in the genotype space gives the total consumption of the resource. We choose *D* = 5·10^−7^ and *γ* = 0.11 in the following simulations.

As the feeding manner, we choose a continuous supply and additions with periodic pulses
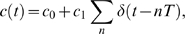
(4)where *δ* is the Dirac delta function. The periodic additions represent the input fluctuation. In this paper we choose *T* = 5. We checked that the following results are reproduced over a wide range of the period. Another parameter, *λ*, is introduced to describe the intensity of the fluctuation. The amount of supplied resource is given as *c*_0_ = 1−*λ* and *c*_1_ = *λT*, where the total amount of supply in a period is fixed.

Since we introduced the continuous genotype space, the population is distributed in the space, and the genotype and species do not hold a one-to-one correspondence. Then we use the term quasi-species [20] for a group of the population which has a unimodal distribution in the genotype space. A quasi-species is represented by a genotype which is located at the peak of the distribution. If the mutation rate is sufficiently small, a population of one quasi-species is approximately replaced by the population of the representative genotype.

In the simulations, the genotype space is discretized into a lattice and the fourth order Runge-Kutta method is used, with step size 0.001 and spatial mesh size 0.0045. Population densities less than a threshold 10^−8^ are replaced with zero, which denotes the discreteness of the population. We checked that the results are robust against the changes of the step and mesh size, and the threshold of the population discreteness.

## Results

### Simulations

**Figure 2:**
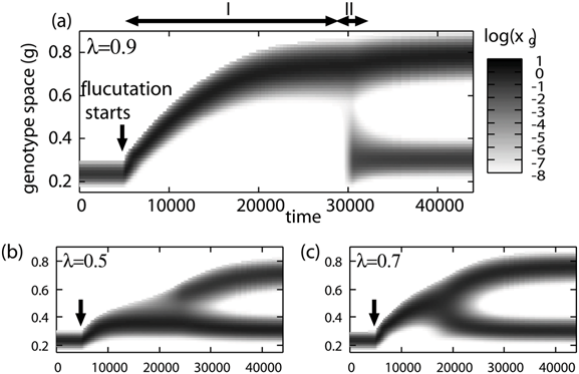
Evolutionary process with periodic fluctuation. Evolutionary processes as the pattern dynamics of population densities in the genotype spaces. The conditions of three different intensities of fluctuation *λ* = 0.5,0.7, and 0.9 are shown. The vertical axis denote the genotype space, and the population density normalized in time with period *T* = 5 is plotted with gray-scale in a logarithmic scale. Initially, static conditions (*c*_0_, *c*_1_) = (1−*λ*,0) are set, and stable genotype compositions are provided. Fluctuations are introduced at *t* = 5000 (*c*_0_, *c*_1_) = (1−*λ*,5*λ*), and evolutionary branching is clearly observed after that.

**Figure 3:**
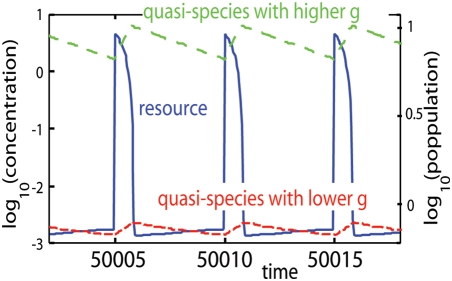
Steady state oscillation. Oscillation of the resource concentration (solid line) and populations of the quasi-species (dashed lines) for *λ* = 0.9. Data are taken after the branching dynamics are completed.

Fig. 2 shows the evolutionary dynamics in the three different conditions, *λ* = 0.5,0.7, and 0.9. In each simulation we initially supply the resource only in a continuous way and the genotype composition of the resident population shows a unimodal distribution in *g* space; i.e., one quasi-species exists. This agrees with the competitive exclusion principle. However, introducing fluctuation in the resource supply (the timings are marked with arrows in Fig. 2), we find dynamic changes of the genotype distributions which lead to evolutionary branching. Each of the emerging branches has a unimodal shape and has no connection with the others. Therefore, each of them is regarded as an independent quasi-species. This indicates that the coexistence of multiple quasi-species is attained. The oscillatory time series of the system after it reaches the final stationary state for *λ* = 0.9 are shown in Fig. 3.

We checked that the evolutionary branching phenomena are robust against changes of model parameters *D*, *γ*, *T* and *c*_0_ *T*+*c*_1_ (total amount of the resource supply in the period). Besides, we also checked the results are robust against the change of the coefficient of the trade-off relation between the parameters 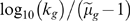; 1/0.3≅3.33 is used in most calculation in this study. For example, with the intensity of fluctuation *λ* = 0.7, the stable evolutionary branching is observed in the range of the coefficient 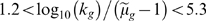.

When the periodic supply is introduced, the genotype distribution changes through two phases, gradual evolution and branching (indicated by I and II in Fig. 2-a). In the first phase, the distribution moves gradually to higher *g* in the genotype space, keeping a unimodal shape, which indicates the gradual evolution of the quasi-species. The branching starts after the movement stops.

**Figure 4:**
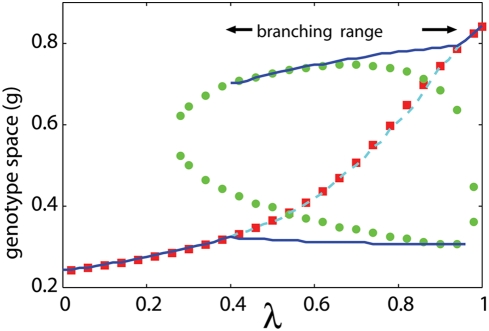
Phase diagram. The dependences of characteristic genotypes on the intensity of fluctuation *λ*. Data from the evolutionary simulations are plotted with lines. The representative genotypes at the end of the first phase are plotted with cyan dashed lines, and the representative genotypes at the final state are plotted with blue continuous lines. Characteristic values of PIPs (discussed below), *g_c_* and *g_ex_*, are plotted with red squares and green circles, respectively. *g_c_* values show good correspondences with genotypes at the end of first phase, *g_ex_* values are related with genotypes at coexisting states.

**Figure 5:**
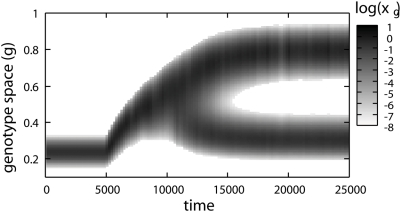
Evolutionary process with stochastic fluctuation. Evolutionary branching induced by a stochastic fluctuation. Instead of using eq. (4), the resource is supplied with the following way: the continuous supply of the resource with 0.2 per unit time is provided through the simulations and the additions with stochastic pulses of Poisson process (averaged number of times of addition per unit time is 0.2 and the amount of adding resource at once is 4.0) starts at *t* = 5000. This simulation indicates that the stochastic fluctuation also induces the clear branching.

The final states of the three figures in Fig. 2 have similar genotype compositions. Therefore, the states with two quasi-species are robust against the intensity of fluctuation *λ*. However, features of evolutionary branching dynamics are different. The moving distances of genotype distributions before the branchings begin vary with the intensity of fluctuation, which is characterized by the positions of distributions in the genotype space at the end of the first phase. In Fig. 4, we plot the genotype representing the quasi-species at the end of the first phase and the genotype(s) representing the quasi-species at the final states against *λ* with lines. Branching was observed in the range *λ* = 0.40∼0.96, where the genotype at the end of the first phase gradually changes while the final genotypes change little. At the no-branching conditions, only the first phase appears, and the two lines coincide. This evolutionary branching is induced not only by the periodic fluctuation but also stochastic fluctuations with Poisson process. In Fig. 5, we give a demonstration of the evolutionary branching in the stochastic environment. It indicates the generality of the branchings induced by fluctuation. Hereafter, we mainly discuss about the system with the periodic fluctuation, because of its simplicity.

Based on the given trade-off relation in parameters of the Monod equation, the finally resident quasi-species are characterized as the specialist adapted for the rich nutrient condition (quasi-species with larger *g*), and as the generalist adapted for wider range of the nutrient condition(quasi-species with smaller *g*). Moreover, quasi-species with larger *g*, which has large growth rate at a specific nutrient condition, is called as an r-strategist in terms of the r/K selection theory, and quasi-species with smaller *g*, which does not decrease its population in wider ranges of nutrient conditions than the other quasi-species, is a K-strategist. Interestingly, the lengths of growing periods and the lengths of decreasing periods are almost the same between the both quasi-species as observed in Fig. 3. Therefore the time-sharing of resource niche [21] does not seem to happen at the coexisting state. Instead, the growth rates and decrease rates in these phases are different from each other: quasi-species with larger *g* has higher growth and decrease rates, and quasi-species with smaller *g* has lower rates.

### Evolutionary invasion analysis

The evolutionary branchings reported here is driven by the competition in population dynamics. It is a frequency-dependent selection process [22], and the evolutionary invasion analysis proposed by Geritz *et al.* [23], [24] are useful to investigate such evolutionary processes. Here, we introduce the technique and analyze our system to describe the processes of evolutionary branchings.

We start the analysis with the non-fluctuating condition, which corresponds to the situations before the periodic fluctuations are added in the three simulations in Fig. 2. We show that there is the only one fittest genotype based on the evolutionary invasion analysis, which is, in this non-fluctuating condition, also called the R* rule [16]. Then, we analyze the fluctuating condition by using the pairwise-invasibility plot (PIP), which is a tool in the analyzing method.

Suppose a system containing one genotype *g* = *α* without mutation and with a non-fluctuating environment. The resource concentration comes to an equilibrium *s̅* *_α_* which holds 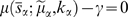 (corresponding to the contour line of zero growth rate in Fig. 1-a). Note that *s̅* *_g_* depends only on the traits of the genotype and is independent from the amount of supply *c*_0_. Thus *s̅* *_g_* is the value characteristic to the genotype. If another genotype *g* = *β* with a smaller equilibrium (*s̅* *_β_*<*s̅* *_α_*) is introduced into the state, the population of *β* grows 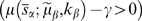. This raises the total consumption of the resource, and the resource concentration decreases. Then the genotype α decreases and the system comes to a new equilibrium state with the population of genotype *β* and the resource concentration *s̅* *_β_*. The monotonic increase of the Monod equation guarantees these processes.

Therefore, the relation between genotypes is determined by the values of *s̅* *_g_*, and the genotype *g_c_* which has the minimum value is the fittest genotype. The resident genotypes are replaced with genotypes with lower equilibriums sequentially until genotype *g_c_* is reached, and the resource concentration comes to 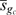. In our model *g_c_* = 0.24 (where the contour line of zero growth rate in Fig. 1-a gives the minimum *s*) which corresponds to the representative genotype of quasi-species at the initial non-fluctuating conditions in simulations.

When fluctuation in the resource supply is introduced, the above simple discussion is not directly applicable. This is because that the growth or decay of populations depends on oscillation patterns of the resource concentration (as an example of the oscillation profile, see Fig. 3). Such a situation is in contrast to the above condition, where only the static value of *s* determines the growth rate. In other words, the variety of oscillation profiles at the fluctuated conditions keeps off from having the simple order relation among genotypes and leads the increase of species diversity. To analyze the fluctuating conditions we introduce the pairwise-invasibility plot (PIP), which gives the relation between genotypes and enables us to describe the evolutionary dynamics with the process of invasion and annihilation.

**Figure 6:**
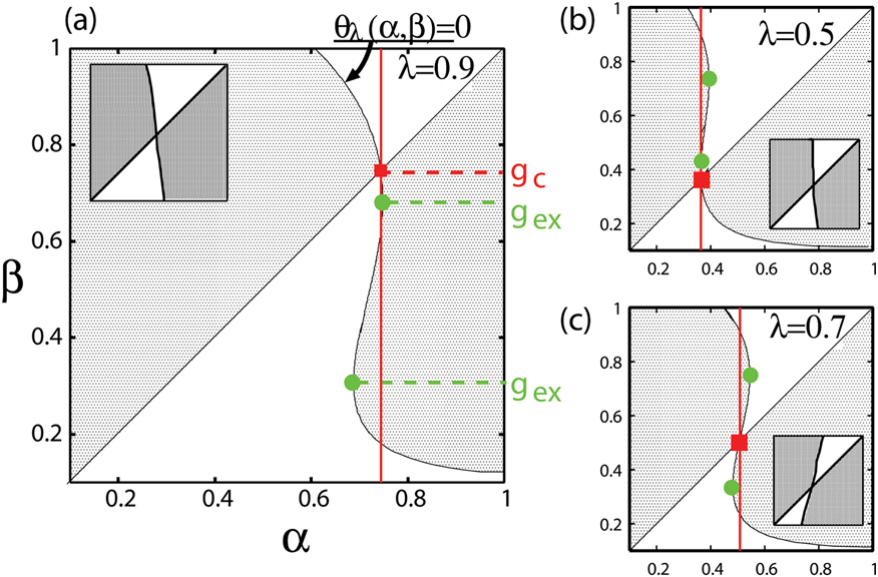
Pairwise invasibility plot. Pairwise invasibility plot for *λ* = 0.5,0.7, and 0.9 (corresponding to Fig. 2). *g_c_* and *g_ex_* are plotted with red squares and green squares, respectively. Vertical lines on *g_c_* (red solid lines) pass through the shaded regions, which denotes genotypes invasible to *g_c_*. Each inset represents the blowup of the corresponding PIP around *g_c_*, which shows the sign of the gradient of *θ_λ_*(*α*, *β*) = 0 at *g_c_*.

PIP is constructed with the following procedure. Suppose a system with one genotype *g* = *α*, which is given by
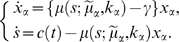
(5)The integration of eq. (5) gives a periodic oscillation function of resource concentration *s̅* *_α_*(*t*) with period *T* after some relaxation time. If a small population of another genotype *g* = *β* is introduced into the system, the average growth rate of *β* is calculated by
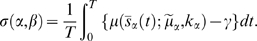
(6)The sign of *σ*(*α*, *β*) determines the invasibility of *β* into the *α* population. Calculating *σ*(*α*, *β*) for all pairs of genotypes and shading the areas of positive *σ*(*α*, *β*) on the *α*-*β* plane, PIP is obtained. The PIPs corresponding to Fig. 2 are shown in Fig. 6.

Here we investigate the structures of the PIPs and use them to illustrate evolutionary dynamics. The PIPs are constructed with two boundary lines. One is the diagonal line *α* = *β*. Since the diagonal line indicates the invasibility to oneself which is neutral (*σ*(*α*, *α*) = 0), it is always the boundary in PIP. The other is a curved line
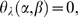
(7)the shape of which determines the characteristics of PIP. First, we refer to the genotype at the intersection between lines as *g_c_* (marked with red squares in Fig. 6). Without fluctuation (*λ* = 0), the intersection coincides with the fittest genotype *g_c_* discussed above. *g_c_* is important because of the following singular property. Any genotype lower than *g_c_* is invaded by genotypes higher than it (area above the diagonal line is shaded) and any genotype higher than *g_c_* is invaded by genotypes lower than it (area below the diagonal line is shaded). This indicates that, as part of the evolutionary process, a resident genotype is invaded and replaced by genotypes closer to *g_c_* one after another. It converges to *g_c_*. Therefore *g_c_* is called the convergent stable genotype [22]. The convergent process corresponds to the gradual evolution of the quasi-species at the first phase in simulations. It stops when the representative genotype agrees with *g_c_*. The agreement between them is shown in Fig. 4.

In Fig. 6, the vertical lines on *g_c_* s pass through the shaded regions. This means that there are genotypes invasible for the population of *g_c_*, and this invasibility promotes evolutionary branching. At the end of the first phase the population distributes around *g_c_* in the genotype space. If the tail of the distribution covers the region of invasible genotypes, the population of these genotypes grow and form another branch. Therefore, the evolutionary branching succeeds as the second phase.

The features of branching dynamics are characterized by the relation between *g_c_* and genotypes invasible to *g_c_*. In PIP for *λ* = 0.5 genotypes higher than *g_c_* are invasible to *g_c_*, both higher and lower genotypes are invasible to *g_c_* for *λ* = 0.7, and lower genotypes are invasible to *g_c_* for *λ* = 0.9. Correspondingly in Fig. 2, the upper branch is born from the lower one for *λ* = 0.5, the unimodal shape separates symmetrically for *λ* = 0.7, and the lower branch is born from the higher one for *λ* = 0.9.

In order to give the evolutionary branching, it is necessary for the line *θ_λ_*(*α*, *β*) = 0 to have three values at *α* = *g_c_*. It is judged by the presence of extremal values of *β* in the line. Thus, the presence and position of the extreme values inform us about the bifurcation properties of the system. We calculate *g_ex_*, genotypes which give the local maximum or minimum for *β* in the line *θ_λ_*(*α*, *β*) = 0 (see Fig. 6),
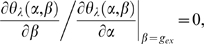
(8)and plot in Fig. 6. *g_ex_* appears in pairs in the range 0.28≤*λ*≤0.98. Particularly, they disappear in small fluctuation range (*λ*≤0.28), the fluctuation gives the three-valued property in this system. At the onset of the branching range, one quasi-species coincides with *g_c_* and the other coincides with *g_ex_*. This is because that at the onset, the curved boundary line *θ_λ_*(*α*, *β*) = 0 is tangent to the vertical line on *g_c_* at *β* = *g_ex_*, and an additional quasi-species arises at the tangent point.

The mechanisms to induce the branching behaviors are mathematically classified into two types based on the gradient of the line *θ_λ_*(*α*, *β*) = 0 at *g_c_*. If the gradient is positive (corresponding to Fig. 6-c), genotypes slightly larger and slightly smaller than *g_c_* are invasible to *g_c_*. Therefore *g_c_* is locally unstable and the branching proceeds independent form the magnitude of the diffusion in genetic space *D* and the threshold of the population discreteness. On the other hand, if the gradient of the line *θ_λ_*(*α*, *β*) = 0 at *g_c_* is negative (corresponding to Fig. 6-a,b), genotypes closely related with *g_c_* are not invasible to *g_c_*, and the *g_c_* is locally stable (locally ESS). In these cases, genotypes distant from *g_c_* are invasible, and whether they invade or not depends on the values of *D* and the threshold of the population discreteness. If *D* is large enough and the threshold is small enough, the tail of the genotype distribution centered at *g_c_* brings the invasible genotypes, which invade and make another branch. Since the sign of the gradient changes at *g_ex_* s, the relation between *g_c_* and *g_ex_* s tells us which types of branching occurs. If *g_c_* has the value intermediate between both *g_ex_* s (corresponding to Fig. 6-c), the gradient of the line *θ_λ_*(*α*, *β*) = 0 at *g_c_* is positive, and the branching proceeds independent from the value of *D* and the threshold of the population discreteness. On the other hand, if the *g_c_* is larger or smaller than both *g_ex_* s (corresponding to Fig. 6-a,b), branching driven by diffusion occurs. The figure 4 indicates the ranges of fluctuation intensity where the *g_c_* is locally unstable and the evolutionary branching occurs independent from *D* and the threshold of the population discreteness: 0.54<*λ*<0.86.

## Discussion

In summary, evolutionary branching dynamics induced by environmental fluctuation is reported in a model of ecosystems competing for a single resource. The evolutionary invasion analysis is applied to illustrate the evolutionary dynamics. Previous studies [10], [11] have reported the coexistence of two species with given parameters in the presence of fluctuation. However, the occurrence of the evolutionary branching as the response to the environmental fluctuation and the evolutionary stability of the coexisting state have remained open questions. Here, we give clear demonstrations of them. The results also supports the general discussions which propose that the temporal fluctuation of environment increase the species diversity of the ecological systems [6], [7].

Although only a case with a periodic fluctuation with the period *T* = 5 is analyzed in this paper, the results are reproduced over a wide range of the period. Moreover, we checked that some stochastic fluctuations with Poisson process in resource supply also induce the evolutionary branching and coexistence. It indicates the generality of the branching reported here.

Here we use a model of a microbial ecosystem to show the evolutionary branching induced by the environmental fluctuation. However, these phenomena are not specific to the model of microbial systems. In the following, we discuss the general mechanisms how the environmental fluctuation increases the species diversity.

MacArthur and Levins [25] indicated that the number of independently adjustable environmental parameters corresponds to the maximum number of coexisting species. Applying it to our system in the non-fluctuating condition, resource concentration is only the adjustable parameter, and it leads to the existence of only one quasi-species. However, the stable co-existence of two quasi-species is seen in cases where fluctuation is present. This suggests that the number of adjustable parameters is increased by introducing environmental fluctuation. When the system is always in an oscillatory state, whether one can grow or not depends on the comprehensive details of the oscillation profile of the parameter. In other words, the whole oscillation profile is the environmental state to be adjusted. The variety of the profile can be described with several dimensional parameters which forms a subspace of a functional space. In this way, introducing fluctuation expands the dimensions of adjustable environmental parameters, and it enables the branching and the coexistence of genotypes with different strategies.

**Figure 7:**
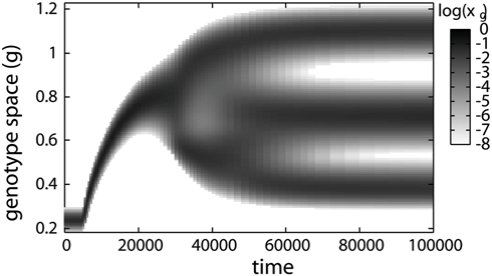
Evolutionary process with bi-periodic fluctuation. Evolutionary branching and the resulting three quasi-species coexistence induced by a bi-periodic fluctuation in environment. We choose resource supplying function *c*′(*t*) which is constructed by two different periodic function with periods 5.9 and 50, *c*′(*t*) = 0.03+0.4Σ*δ*(*t*−5.9*n*)+500Σ*δ*(*t*−50*n*). The fluctuation starts at *t* = 5000. After that and the system shows transient branching dynamics and reaches the three quasi-species coexistence state.

The diversity of oscillation profiles is not restricted to two-dimensional parameter space. Thus, by the principle, the coexistence of more than two species is possible. For example, a three-species coexistence easily realizes if one changes the environmental fluctuation from mono-periodic to bi-periodic (Fig. 7). In addition, the environmental fluctuation is not necessary to be input in above discussion. The evolutionary branching induced by the self-sustained oscillation in population dynamics [14], [15] and the branching reported here can be understood by unified mechanisms.
